# Probable Leptospirosis in the Adventure Traveler with Freshwater Exposure: Narrative Review and Case Series

**DOI:** 10.3390/pathogens15070677

**Published:** 2026-06-26

**Authors:** Gregory D. Hawley, Ambika Agrawal, Maryam Alhashmi, Milica Novakovic, Andrea K. Boggild

**Affiliations:** 1 Temerty Faculty of Medicine, University of Toronto, Toronto, ON M5S 3H2, Canada; 2 Tropical Disease Unit, Toronto General Hospital, Toronto, ON M5G 2C4, Canada; 3 Institute of Medical Science, University of Toronto, Toronto, ON M5S 3H2, Canada

**Keywords:** adventure travel, fever in the returned traveler, kayaking, leptospirosis, whitewater rafting, zoonoses

## Abstract

Leptospirosis is an emerging zoonotic infectious disease in the adventure traveler with recreational freshwater exposure. We describe three cases of probable leptospirosis acquired during whitewater rafting trips in rural Ecuador. Rafting took place following periods of heavy rainfall and flooding of rivers, which are well documented risk factors for leptospirosis. Two of our patients presented to our rapid assessment clinic with acute febrile illness after travel to a region with a confirmed leptospirosis outbreak, while the third patient presented four weeks after a convalescent episode of acute febrile jaundice during a dramatic rise in reported leptospirosis cases in Ecuador. Delayed turnaround times for confirmatory microbiologic diagnostics in leptospirosis challenge its management and necessitate provisional clinical diagnosis and empiric antimicrobials based on compatible clinical symptoms, biochemical abnormalities, exposure history, and known outbreak epidemiology. We herein situate the presentations of our patients within the broader literature by reviewing the clinical, diagnostic, therapeutic, and prognostic considerations when caring for leptospirosis patients. Leptospirosis should be considered in the differential diagnosis of all patients presenting with acute febrile illness following adventure travel with freshwater exposure, and empiric treatment should be considered given the absence of readily available confirmatory microbiologic testing.

## 1. Introduction

Leptospirosis is a ubiquitous zoonotic infectious disease that accounts for over one million cases and 58,900 deaths annually across the globe [1]. Leptospirosis is caused by a spirochete infection from the genus *Leptospira*, consisting of multiple pathogenic serovars [2,3,4,5]. Leptospirosis manifests across a wide range of symptomatology, from asymptomatic infection to mild nonspecific febrile illness to severe infection with resultant multiorgan failure and death [1,2,3,4,6,7]. Weil’s disease, the most feared manifestation of leptospirosis, is characterized by the triad of jaundice, renal failure, and hemorrhage typically following resolution of the acute primary infection and signified by onset of relapsing fever in a biphasic illness [8,9].

*Leptospira* reproduction occurs in the renal tubules of infected mammalian hosts such as rodents and canines, thereby leading to urinary excretion of leptospires and subsequent environmental contamination of soil and water [1,2,4,6,7,10]. Transmission of leptospirosis can thereby occur following direct contact with infected animals or indirect contact with contaminated water and soil [1,2,3,4,5,6,10,11,12,13]. Leptospirosis remains an occupational hazard for industries in close contact to soil and water systems (sewage workers, waste workers, farmers, aquaculture workers, military personnel, and construction workers) or frequent animal contact (livestock attendants, abattoirs, veterinarians, hunting and game managers, animal control workers, and scientists) [1,2,3,4,5,6,11,12,13,14,15,16,17,18,19]. High-density populations of low socioeconomic status are disproportionately affected by leptospirosis due to inadequate sanitation, lack of safe drinking water, and proximity to infected animals [1,6,13,17,20,21,22]. As such, leptospirosis continues to be a disease of poverty with differential impacts for those living in the “global South”, particularly areas of severe resource constraint and regions vulnerable to climatologic extremes such as tropical storms, cyclones, and flooding [23,24]. The year 2023 saw a large outbreak of leptospirosis in coastal areas of Ecuador [25], with hospitalizations due to leptospirosis four times higher in 2023 than in the years 2018–2022 [26].

Leptospirosis is an emerging zoonotic infectious disease in adventure travelers due in part to contact with contaminated waters during recreational activities. We herein describe three cases of probable leptospirosis acquired during whitewater rafting trips in rural Ecuador and situate their presentations within the broader literature by reviewing the clinical, diagnostic, therapeutic, and prognostic considerations when caring for leptospirosis patients. The aim of this case series is to review the clinical and diagnostic considerations when evaluating suspected leptospirosis, in addition to conducting a literature review on an emerging source of leptospirosis, recreational water exposure in adventure travelers.

## 2. Methods

### Diagnostic Classification

Leptospirosis diagnoses were classified based on 2025 case definitions provided by the U.S. Centers for Disease Control and Prevention (CDC) [27]. A diagnosis of leptospirosis may be classified as confirmed or probable based on a combination of clinical criteria, laboratory criteria, and epidemiologic linkage. A probable case is defined as a diagnosis of leptospirosis that meets clinical criteria and presumptive laboratory evidence, or that meets clinical criteria and epidemiologic linkage criteria. A confirmed case is defined as a diagnosis of leptospirosis underpinned by confirmatory laboratory evidence.

## 3. Clinical Cases

Here, we describe three cases of probable leptospirosis (Table 1) acquired via recreational freshwater exposure during two separate whitewater rafting trips to Ecuador. The first two patients were previously healthy men who presented to the Emergency Department (ED) at a tertiary care academic hospital in Toronto with symptoms of fever, fatigue, myalgia, nausea, and vomiting following a sixteen-day whitewater rafting trip to Ecuador. One year later, a previously healthy female presented to the same ED following a six-week whitewater rafting trip to Ecuador, during which she developed a self-limited illness with fever and jaundice that necessitated inpatient admission in Ecuador. All three cases were associated with high-risk freshwater exposures during periods of heavy rainfall and flooding, consistent with known environmental risk factors for leptospirosis.

### 3.1. Patient A

Patient A, a Canadian resident in his 50s originally from Ireland with no past medical history, developed symptom onset five days after returning from a whitewater rafting trip in Ecuador. He endorsed sudden onset fatigue, followed by fever (40.8 °C), nausea, vomiting, abdominal pain, arthralgia, and myalgia. After three days of fever, he presented to the ED at our site, where he was febrile (38.5 °C) and hemodynamically stable. Initial laboratory investigations revealed thrombocytopenia (103 × 10^9^/L; reference range 150–400 × 10^9^/L), lymphopenia (leukocytes 6.4 × 10^9^/L; reference range 4.0–11.0 × 10^9^/L, lymphocytes 0.2 × 10^9^/L; reference range 1.5–4.0 × 10^9^/L), elevated serum creatinine (129 μmol/L with no baseline; reference range 50–98 μmol/L), and biochemical hepatitis (alanine aminotransferase [ALT] 38 U/L; reference range < 40 U/L, bilirubin 41 μmol/L; reference range < 22 μmol/L). A chest radiograph demonstrated small bilateral pleural effusions. Malaria screen and respiratory virus nasopharyngeal swab polymerase chain reaction ([PCR] targeting influenza A/B, respiratory syncytial virus [RSV], and SARS-CoV-2) were negative. Blood cultures, dengue IgM, and Chikungunya IgM were sent. He received intravenous fluids and was discharged home with a referral for a next-day appointment in our clinic.

When seen for consultation in our clinic, the patient was afebrile and hemodynamically stable. Physical examination demonstrated bilateral conjunctival injection and a diffusely tender abdomen without signs of peritonitis. The remainder of his physical examination was within normal limits. His travel history was notable for a sixteen-day camping and whitewater rafting trip in freshwater rivers of rural Ecuador. He reported that flash floods occurred throughout their travel after periods of heavy rainfall, which caused water levels in the rivers to rise. He reported that the rising river levels created a more enjoyable rafting experience and that rafting took place frequently on recently flooded rivers. He swam in the rivers and waded in knee-deep water for multiple hours each day. He recalled minor abrasions to the lower extremities during rafting. He recalled that the rivers were located adjacent to farmland. The trip also included multiple hikes through the jungle. He denied consumption of raw fish or meat, or unpasteurized dairy products. He endorsed exposure to domestic dogs and frequent mosquito bites but denied other animal or arthropod contact. He denied new sexual partners or illicit substance use. He did not receive pretravel consultation and did not take any form of chemoprophylaxis. He reported that two other travelers experienced self-limited gastrointestinal symptoms during the trip that did not require medical attention.

The day prior to assessment in our clinic, there was a press report of a confirmed leptospirosis outbreak in rural Ecuador following heavy rains and flooding [28]. Given his clinical symptoms, freshwater exposure, thrombocytopenia, renal dysfunction, abnormal liver enzymes with a predominant cholestatic picture, and epidemiologic linkage via an exposure event associated with a confirmed leptospirosis outbreak, he was clinically and provisionally diagnosed with probable leptospirosis and started on oral doxycycline. Same-day laboratory investigations were notable for proteinuria (1 g/L).

The patient returned to the ED later that evening due to intolerance of oral doxycycline and was admitted to the General Medicine service. Infectious Diseases was consulted and started intravenous therapy with 2 g of ceftriaxone daily. During his admission, he developed bilateral calf pain and right upper quadrant pain. His platelets declined to a nadir of 78 × 10^9^/L, with normocytic anemia (Hb 123 g/L; reference range 140–180 g/L), biochemical hepatitis with bilirubin predominance (bilirubin 70 μmol/L), and elevated serum creatinine (123 μmol/L). An abdominal ultrasound was notable for hepatosplenomegaly. Following two doses of intravenous ceftriaxone, he defervesced and was able to tolerate oral intake. His platelets improved to 98 × 10^9^/L, his bilirubin was downtrending (33 μmol/L), and his acute kidney injury resolved. He was discharged with five days of oral doxycycline to complete a total of seven days of antimicrobial therapy and followed up in our clinic.

Over the next two weeks, Patient A had complete resolution of his clinical symptoms and biochemical abnormalities. Acute *Leptospira* serology drawn on day five of illness was negative, but convalescent serology collected four weeks later returned positive for *Leptospira* IgM. A microscopic agglutination test (MAT) was negative. Patient A met the CDC case definition for probable leptospirosis based on clinical criteria, presumptive laboratory evidence, and epidemiologic linkage criteria [27]. Additional microbiologic investigations, including malaria screen, blood cultures, dengue IgM, and viral hepatitis serologies, were negative. A repeat ultrasound at four months showed resolution of hepatosplenomegaly.

Patient A returned to Ecuador the subsequent year for another whitewater rafting trip and was provided a prescription for leptospirosis chemoprophylaxis with 200 mg of doxycycline once weekly while traveling. He was adherent to the chemoprophylaxis regimen without any febrile illness associated with this subsequent travel nor any adverse effects from the doxycycline.

### 3.2. Patient B

Patient B, a Canadian resident in his 20s with no significant past medical history, developed fever (39.4 °C), fatigue, myalgia, nausea, and vomiting six days following his return from the same whitewater rafting trip in Ecuador as Patient A. He first presented to a community ED, where he was afebrile and hemodynamically stable. His complete blood count was notable for leukocytosis (14.3 × 10^9^/L) with neutrophilia (12.9 × 10^9^/L; reference range 2.0–7.5 × 10^9^/L) and lymphopenia (0.4 × 10^9^/L). He had mildly elevated bilirubin (20 μmol/L; reference range 3–17 μmol/L), hyponatremia (134 mmol/L; reference range 136–145 mmol/L), hypomagnesemia (0.69 mmol/L; reference range 0.74–0.99 mmol/L), and hypophosphatemia (0.71 mmol/L; reference range 0.81–1.45 mmol/L). He was diagnosed with traveler’s gastroenteritis and treated with intravenous fluids, magnesium supplementation, and anti-emetics before he was discharged home. He called his family doctor the following day due to ongoing symptoms and was advised to come to the ED at our center. In our ED, he was again afebrile and hemodynamically stable, with a heart rate of 99 bpm. Laboratory investigations drawn in the ED showed ongoing neutrophilia and lymphopenia (leukocytes 10.2 × 10^9^/L, neutrophils 9.4 × 10^9^/L, lymphocytes 0.3 × 10^9^/L). Bilirubin was normal (16 μmol/L; reference <22 μmol/L), with interval development of a mildly elevated ALT (44 U/L). Renal function was normal (creatinine 67 μmol/L). Microbiologic investigations were notable for SARS-CoV-2 virus detection by nasopharyngeal swab PCR. Malaria testing by Giemsa-stained thick and thin smear microscopic examination and rapid antigen testing (BinaxNOW, Abbott, Abbott Park, IL, USA) was negative. Blood cultures, dengue IgM, viral hepatitis serologies, and urine chlamydia/gonorrhea nucleic acid amplification tests (NAATs) were ordered. He was given intravenous fluids, ondansetron, and acetaminophen and discharged home with follow-up in our clinic.

When seen for consultation in our outpatient clinic the following day, Patient B reported interval onset of watery, non-bloody diarrhea and headache. He continued to have non-bloody emesis and endorsed persistent myalgia and arthralgia, predominantly in the knees, low back, and neck. He denied oropharyngeal, respiratory, cardiac, genitourinary, or dermatologic symptoms. He recalled a similar illness with fever, myalgia, chills, nausea, and vomiting while traveling in Ecuador that resolved spontaneously after three days. On examination, he was febrile (38.3 °C) and tachycardic (119 bpm). There was no jaundice, icterus, or conjunctival suffusion. He had mild erythema of the oropharynx and a small palpable, subcentimeter cervical lymph node. The remainder of his physical examination was within normal limits.

Detailed travel history elicited the same sixteen-day camping and whitewater rafting trip as Patient A, where both individuals had frequent exposure to recently flooded rivers for whitewater rafting, swimming, and wading in knee-deep water. Patient B also recalled sustaining minor abrasions to the lower extremities during rafting. He similarly denied consumption of raw fish or meat, or unpasteurized dairy products while in Ecuador, but did have sushi from a restaurant in Canada the day prior to symptom onset. He also reported exposure to domestic dogs and mosquito bites, but denied other animal or arthropod exposure. He denied sexual contact or illicit substance use. He did not receive pretravel consultation, nor did he take any form of chemoprophylaxis.

In the setting of his biphasic febrile gastrointestinal illness, neutrophilia, and freshwater exposure while rafting, a primary differential diagnosis of leptospirosis versus bacterial traveler’s gastroenteritis was proposed, in the context of concurrent SARS-CoV-2 exposure. He was prescribed a course of ciprofloxacin for gastroenteritis (500 mg orally twice daily for three days) with next day follow-up. When seen the following day, he reported ongoing fever and frequent watery, non-bloody diarrhea (>30 bowel movements in 24 h). He continued to have headache, posterior neck pain, myalgia, and arthralgia. He was again febrile (40.2 °C) and tachycardic (114 bpm). Interval investigations showed proteinuria (0.3 g/L), anemia (hemoglobin 128 g/L), leukocytosis with neutrophilia, and elevated bilirubin (23 μmol/L), with the remainder of his liver enzymes within normal limits. That morning, the aforementioned press report of a confirmed outbreak of leptospirosis in rural Ecuador following heavy rains and flooding was published [28]. Based on his extensive recent freshwater exposure in a confirmed outbreak region, lack of clinical improvement, and laboratory findings of proteinuria, leukocytosis, and elevated bilirubin, he was started on 100 mg of oral doxycycline twice daily for suspected leptospirosis. His stool culture later returned positive for *Campylobacter jejuni/coli*, for which he continued ciprofloxacin. Given his strong epidemiologic linkage consisting of multiple exposures in a confirmed outbreak region, clinical syndrome, and laboratory findings including elevated bilirubin and proteinuria, his clinical picture was not felt to be explained by the positive stool culture and SARS-CoV-2 nasopharyngeal PCR alone, and was as such continued on the course of doxycycline for suspected leptospirosis.

The patient had significant clinical improvement within 24 h of initiating doxycycline therapy. At the end of his treatment course, he had complete resolution of his clinical symptoms and biochemical and hematological abnormalities. Microbiologic investigations were negative for bacteremia, dengue, *Strongyloides*, *Schistosoma*, *H. pylori*, acute or chronic hepatitis B infection, hepatitis C, stool ova and parasites, and urine chlamydia and gonorrhea. His acute *Leptospira* serology (drawn on day four of symptoms) was negative. The patient was given requisitions for convalescent *Leptospira* serology at multiple follow-up visits but did not complete this bloodwork. Nevertheless, given his recreational freshwater exposure in a confirmed outbreak region, clinical and biochemical syndrome, and response to therapy, his picture was consistent with probable leptospirosis (as per CDC case definition based on clinical criteria and epidemiologic linkage) [27] along with microbiologically confirmed *Campylobacter* gastroenteritis and COVID-19 exposure.

### 3.3. Patient C

Approximately one year after the previous cases, Patient C, a Canadian resident in her 20s with no past medical history, presented to the ED on the day of her return from Ecuador with abdominal pain, loose stools, and a small gynecological mass. She was afebrile and hemodynamically stable in the ED. Physical examination noted mild epigastric tenderness on examination. She had a normal complete blood count and renal function, with mild derangements in her liver enzymes (ALT 62 U/L, AST 41 U/L; reference range 5–34 U/L, bilirubin 29 μmol/L). She was referred to our clinic and seen the following day, by which time she had resolution of her gastrointestinal symptoms.

During her consultation, Patient C reported multiple acute illnesses while participating in a nearly six-week long whitewater rafting expedition across rural Ecuador. She first developed an acute gastrointestinal illness several days into her trip, characterized by 5–6 episodes of non-bloody diarrhea per day, without accompanying fever or systemic symptoms. She was seen by a local provider and prescribed secnidazole, albendazole, and a ten-day course of ciprofloxacin. Despite antimicrobial therapy, she worsened a few days later with the development of fever and jaundice. She sought medical care at a hospital in Ecuador and was diagnosed with acute liver failure. She did not recall receiving a formal diagnosis for her liver failure, and no microbiologic investigations were available from that time. She reported receiving intravenous hydration but no further antimicrobial therapy. She was discharged and had complete resolution of her jaundice over the course of 1–2 weeks. She then developed symptoms of dysuria, hematuria, and lower abdominal pain. She saw a doctor at a local clinic and was diagnosed with a urinary tract infection, for which she was treated with nitrofurantoin, resulting in complete symptom resolution. At this point, she was feeling well and returned to rural Ecuador to continue rafting. Several weeks later, she developed acute onset high-grade fever and again sought emergency medical care in Ecuador. She reported that laboratory investigations were positive for thrombocytopenia and dengue fever, although the method of microbiologic testing for dengue was unavailable for reference. She was admitted for monitoring, during which time she reported fever and generalized body rash for five days. She was discharged with resolution of her fever and rash.

Her exposure history was significant for near-daily whitewater rafting while clinically well throughout most of her six-week stay in Ecuador. She reported that her group rafted frequently after heavy rains, when river levels were high. She recalled that her raft flipped over multiple times with submersion underwater but did not recall any readily apparent abrasions or local skin trauma. She denied direct contact with animals or animal urine. She denied consumption of raw or undercooked meat or seafood, or unpasteurized dairy products. She drank bottled water but did consume ice of unknown origin. She had one long-term monogamous partner in Canada and denied new sexual contacts. She did not receive pretravel consultation, nor did she take any form of prophylaxis. She did, however, specifically recall receiving a full series of hepatitis A and B vaccines (Twinrix) in Canada approximately 10 years prior for other international travel.

Microbiologic testing performed at her initial visit, which was over four weeks after her acute febrile illness with jaundice, revealed reactive *Leptospira* IgM. MAT was negative. Additional microbiologic testing was notable for reactive hepatitis A IgG, non-reactive hepatitis B surface antigen, negative blood cultures, negative malaria screen, and negative serologies for hepatitis C, dengue (IgM and IgG), HIV, syphilis, and *Strongyloides*. Follow-up liver enzymes revealed normalization of ALT and AST with persistent mild hyperbilirubinemia (bilirubin 32 μmol/L). The patient later endorsed a long-standing history of asymptomatic mildly elevated bilirubin. She remained clinically well thereafter.

Patient C’s clinical presentation was consistent with probable leptospirosis based on clinical criteria and presumptive laboratory evidence of a positive *Leptospira* IgM. Although the patient did not have a definitive epidemiologic linkage to a confirmed outbreak, there were multiple reports of increased leptospirosis cases in Ecuador in the first six months of 2025 [29,30], which coincided with her repeated freshwater exposure during rafting. The patient clearly recalled rafting during periods of heavy rainfall and river flooding, which were factors that attributed to the rise in cases in 2025 [29,30]. Although the patient’s microbiologic testing was also positive for reactive hepatitis A IgG, which is another common cause of acute febrile jaundice in the traveler, she clearly recalled receiving two doses of hepatitis A vaccination (Twinrix) in a pretravel consultation several years prior for travel to Southeast Asia. Although the lack of available records from her inpatient admission in Ecuador precludes the complete exclusion of other etiologies, her initial clinical syndrome of acute, self-limited febrile jaundice, presumptive laboratory evidence with reactive *Leptospira* IgM, and strong epidemiologic risk factor of multiple rural freshwater exposures in a country with a dramatic rise in leptospirosis cases support a diagnosis of probable leptospirosis.

## 4. Discussion and Implications for Clinical Care

Leptospirosis is an emerging zoonotic infectious disease in the adventure traveler with recreational freshwater exposure. Recently, there has been increasing recognition of transmission of leptospirosis during recreational and adventure sports with exposure to freshwater, as in the case of our three rafters. A number of reports of leptospirosis following whitewater rafting and kayaking are described in the medical literature [31,32,33,34,35,36,37,38,39,40,41,42,43]. Leptospirosis cases have also been linked to recreational kayaking and canoeing [15,40,42,44,45,46,47,48,49,50,51,52]. Although predominant in tropical countries, with most cases reported from Thailand, outbreaks have been described in temperate regions such as France, Germany, Ireland, the United Kingdom, and the United States of America [15,31,32,33,34,35,36,37,38,39,40,41,42,43,44,45,46,47,48,49,50,51,52]. Risk factors associated with leptospirosis include ingestion of river or lake water, being submerged underwater, and sustaining abrasions or cuts during freshwater exposure [32,33,34,40,41]. Multiple cases reported antecedent heavy rains and/or flooding [15,33,36,39,41,42,46,48]. The rise in popularity of endurance races, such as triathlons, Iron Man competitions, and various ecological races, has coincided with leptospirosis outbreaks in these settings [51,53,54,55,56,57,58,59,60,61,62,63,64]. Other adventure sports with exposure to freshwater, including canyoning, caving, and river surfing, have also been implicated in cases of leptospirosis [50,65,66,67,68].

Our cases highlight three adventure travelers with freshwater exposure (whitewater rafting) in a tropical environment following heavy rains and flooding, during which time an active outbreak of leptospirosis was ongoing. All individuals had extensive freshwater exposure in addition to risk factors such as abrasions to the lower extremities or submersion underwater while rafting. In the setting of a febrile illness with exposure to freshwater adventure sports in a tropical environment, a number of infectious etiologies must be considered (Table 2) [69,70,71,72,73]. These include leptospirosis; traveler’s diarrhea due to commonly reported bacterial enteropathogens (such as *Salmonella* spp., *Shigella* spp., *Campylobacter* spp., and *E. coli*), less commonly reported bacterial enteropathogens (such as *Aeromonas* spp. and *Pleisiomonas shigelloides*), viruses (such as norovirus, rotavirus, and hepatitis A virus), and protozoa (such as *Cryptosporidium* spp., *Giardia intestinalis*, and *Entamoeba histolytica*); acute schistosomiasis; and, under exceptionally rare circumstances, illnesses such as tularemia, melioidosis, and free-living amoebiasis. Dermatologic findings should prompt consideration of less frequently encountered pathogens such as non-tuberculous mycobacterial infections and *Vibrio vulnificus*. Although *Vibrio vulnificus* is halophilic, travelers with recreational freshwater exposure may also have exposure to brackish waters, especially during times of heavy rains and flooding in coastal regions, and as such should remain on the differential even without definitive saltwater exposure. Travelers may also be at risk of infections acquired from soil or organic matter alongside riverbanks, such as mycobacterial infections and endemic mycoses. In the case of patients A and B, who both presented acutely unwell with non-specific febrile illness, hematological derangements, proteinuria, and mild cholestatic hepatitis following travel to Ecuador, malaria, arboviral infection such as dengue, rickettsiosis, and typhoid fever remained high on the differential, especially in the absence of chemoprophylaxis or mosquito-prevention measures. The patients’ non-specific febrile illness in the setting of recreational freshwater exposure, with proteinuria and biochemical hepatitis with bilirubin predominance, placed leptospirosis at the forefront of our differential diagnosis, particularly given the biphasic nature of illness in Patient B and the rapidly evolving hepatorenal syndrome in Patient A.

The first-line antimicrobial treatment of leptospirosis is typically oral doxycycline, with alternatives including penicillin and azithromycin [69]. In patients with severe disease or those unable to tolerate oral intake, inpatient admission for intravenous antibiotics is warranted. In the case of Patient A, ceftriaxone was started until he was able to tolerate oral intake and step-down to doxycycline. Ampicillin, penicillin G, and doxycycline, where available, are alternative intravenous options for leptospirosis [74]. For travelers with an itinerary that includes frequent freshwater exposure, particularly in the context of recreational activities or adventure sports, leptospirosis chemoprophylaxis should be considered. Chemoprophylaxis with doxycycline 200 mg once weekly was shown to be 95% effective in preventing leptospirosis infection with short-term exposure to endemic areas [75]. In a retrospective analysis of a leptospirosis outbreak stemming from an Eco Challenge race in Borneo, pre-exposure prophylaxis with doxycycline was found to be protective against leptospirosis [55]. Travelers receiving doxycycline chemoprophylaxis should be counseled on the side effects of doxycycline, including antibiotic-associated diarrhea, nausea, vomiting, erosive esophagitis, and photosensitivity. Pretravel consultation should include an individualized assessment of leptospirosis risk factors, with discussion of doxycycline chemoprophylaxis for those with high-risk exposures such as rafting in endemic countries.

This case series presents a number of teaching points around the diagnosis and management of leptospirosis in a low-incidence setting. First, the differential diagnosis of fever in a returning traveler should be kept broad in the face of positive microbiologic testing that does not readily explain the patient’s clinical condition. Despite a positive SARS-CoV-2 nasopharyngeal swab in the ED and subsequent culture of *Campylobacter jejuni/coli* in the stool of Patient B, he continued to worsen symptomatically with biochemical abnormalities—notably elevated bilirubin and proteinuria—not fully attributable to these microbial entities. This prompted further consideration of leptospirosis, at which point the patient recovered quickly upon appropriate treatment with doxycycline. The risk of premature diagnostic closure is high in the face of positive initial microbiological investigations that could explain at least some of a patient’s signs and symptoms. However, just like with the care of ill persons living with HIV [76,77,78], Occam’s razor should be judiciously and cautiously applied in tropical medicine given the common occurrence of co-infections that are increasingly recognized with sophisticated highly sensitive and multiplexed molecular diagnostic platforms [79,80,81,82,83,84,85]. The role of possible coinfections in Patient C provided an even greater challenge due to the lack of available medical records from her acute illnesses in Ecuador. The patient’s reported diagnoses of dengue fever and urinary tract infection could not be corroborated or supported by laboratory or microbiologic testing from Ecuador, nor with convalescent testing in her post-travel assessment, as her dengue serology remained nonreactive several weeks after her acute illness. Certainly, given her multiple distinct illnesses during travel (diarrheal illness, fever and jaundice, lower urinary tract symptoms, fever, and rash), the length of her travel, and the numerous environmental exposures in the absence of chemoprophylaxis or stringent arthropod bite prevention measures, the occurrence of multiple independent illnesses remains likely. Careful evaluation of post-travel illness necessitates the consideration of both coinfections and independent, temporally distinct illnesses, as evidenced by the cases of Patients B and C.

Second, like several other tropical infectious diseases, including but not limited to malaria, brucellosis, relapsing fevers, and typhoid fever, leptospirosis can classically present as a biphasic febrile illness [86], with the second phase potentially signifying onset of the immune icteric phase and progression to Weil’s syndrome. When a second phase of illness occurs, it typically does so after a short period of defervescence (three to four days) and, like the initial phase, is generally characterized by non-specific constitutional symptoms such as fever, chills, myalgia, and headache [9]. Resolution of initial phase symptoms also coincides with production of antibodies [9], and as such, the success of standard serologic testing for leptospirosis (i.e., *Leptospira* IgM EIA) relies on optimal timing of specimen collection according to expected antibody production windows. Age—along with jaundice and acute kidney injury (AKI)—has emerged as an independent risk factor for progression to Weil’s syndrome and death [87]. Our experience with the three cases described herein corroborates this phenomenon in that our oldest patient had the most severe clinical syndrome, characterized by intractable vomiting, thrombocytopenia, elevated bilirubin, and AKI.

Third, the two patients in our series who were tested during the acute phase of illness had negative serology, while the two who were tested during convalescence had detectable *Leptospira* IgM antibodies, as signified by positive *Leptospira* EIAs. However, neither of those patients with positive IgM EIAs produced antibodies detectable by the in-house confirmatory MAT assay used at our National Microbiology Laboratory [88]. While MAT assays are regarded as highly specific, with the added advantage of being able to potentially discriminate amongst serovars, their performance during acute phase illness and their sensitivity, in general, is suboptimal [89,90,91]. While culture-based diagnosis is theoretically possible, this requires prolonged incubation (i.e., 6–16 weeks), correctly timed specimen collection (i.e., within the first week of illness for blood and CSF, and in weeks 2–3 of illness for urine), and specialized media not readily available in most laboratories, and it has poor sensitivity [9]. As such, culture-based diagnosis of leptospirosis was not pursued in any of our patients. As with evolution in diagnostic microbiology, in general, molecular methods, such as polymerase chain reaction (PCR) [92] and even next-generation sequencing [93], are being increasingly utilized in both individual patient-level diagnosis as well as surveillance and control activities.

Fourth, monitoring global patterns of disease outbreaks is crucial to inform diagnostic considerations. A press release detailing the emergence of a leptospirosis outbreak in rural Ecuador was important supporting epidemiology in our diagnostic and decision-making process. This is especially important in cases where, as noted above, microbiologic testing may have reduced sensitivity in early infection and/or face long turn-around times. In the case of Patient A, acute *Leptospira* serology was negative and only returned positive in our convalescent sample four weeks later. Therefore, high clinical suspicion must be maintained even in the face of initial negative serological testing. Empiric treatment based on clinical findings and epidemiological risk should be considered in the absence of diagnostics with rapid, actionable, turnaround times, as highlighted by our cases [86].

### Limitations

This case series highlights several important limitations in the diagnosis of suspected leptospirosis in the febrile returned traveler. IgM EIAs have reduced sensitivity early in the acute phase of infection, and serologic diagnosis often relies on convalescent testing to assess for interval seroconversion and MAT positivity. This requires patients to complete follow-up bloodwork several weeks after initial illness, which they may deem unnecessary or of lower importance in the face of complete clinical recovery, as in the case of Patient B. In addition to suboptimal performance of MAT during acute illness, as discussed above, MAT may also be negative if the infecting strain is not represented in the testing panel of *Leptospira* antigens. For travelers with prolonged travel duration, such as Patient C, acute illness may occur while abroad, and the lack of availability of international medical records necessitates that post-travel providers rely on historical self-reported data and convalescent phase testing alone. As such, none of the described cases meet the criteria for confirmed cases of leptospirosis according to CDC case definitions [27]. Understanding the limitations of readily available reference-level diagnostics and employing a holistic approach to diagnosis of suspected leptospirosis based on exposure history and epidemiologic risk, clinical syndromes, laboratory features, and supporting microbiologic testing, where available, are crucial in order to forego early diagnostic closure in the face of negative or absent microbiologic testing. In the absence of environmental sampling of the precise rafting sites, it is impossible to establish a definitive causal relationship between the rafting exposures and clinical illness; however, the strong epidemiologic linkage to a documented leptospirosis outbreak associated with heavy rainfall and flooding supports this mechanism of transmission for probable leptospirosis.

## 5. Conclusions

Febrile illness in the traveler returning with a history of recreational or adventure freshwater activities should prompt a unique differential diagnosis based on the region of travel and exposure history. Leptospirosis is a global pathogen that should be considered in all such cases. Clues to the diagnosis include freshwater exposure, known history of regional outbreak, risk factors such as heavy rainfall or flooding, biphasic illness, proteinuria, acute renal failure, hepatocellular dysfunction with bilirubin predominance, and thrombocytopenia. Diagnosis is often clinical in the early stages. Serology is the mainstay of microbiologic diagnosis but may be negative in early disease, may be available only at regional or national reference centers, and may take several weeks for results to return. PCR, although more sensitive in early infection, is limited by resource availability. As such, suspected cases should be treated empirically with appropriate antimicrobials instead of awaiting microbiologic confirmation. Clinicians should seek confirmatory microbiologic testing based on local resource availability, including the use of acute and convalescent phase samples, but should be aware of their limitations and not rely exclusively on these tools when making diagnostic and therapeutic decisions. Clinicians should be alert for severe manifestations of leptospirosis including icteric leptospirosis and Weil’s Disease, classically characterized by the triad of jaundice and hepatocellular dysfunction, renal failure, and hemorrhage (including severe pulmonary hemorrhage). Patients with severe disease or inability to tolerate oral intake should be admitted for intravenous therapy. Pre-travel consultation is an important preventative measure, as chemoprophylaxis with doxycycline should be considered for high-risk adventure travelers.

## Figures and Tables

**Table 1:** Overview of clinical cases of probable leptospirosis acquired from whitewater rafting in Ecuador.

Case	Age	Sex	Clinical Syndrome	Laboratory and Imaging Findings	Acute Serology	Convalescent Serology	Diagnostic Classification ^#^	Treatment	Outcome
A	50s	M	Anicteric. Fever, nausea, vomiting, myalgia (calves), arthralgia.	Thrombocytopenia, anemia, acute kidney injury, hyperbilirubinemia, proteinuria. Bilateral pleural effusions (CXR) and hepatosplenomegaly (AUS).	Negative	IgM positive, MAT negative	Probable (clinical criteria + presumptive laboratory evidence + epidemiologic linkage)	Inpatient IV ceftriaxone, step-down outpatient oral doxycycline for total duration of seven days	Complete recovery
B	20s	M	Anicteric. Fever, myalgia, headache, neck pain, nausea, vomiting, diarrhea.	Leukocytosis, neutrophilia, lymphopenia, anemia, hyperbilirubinemia, transaminitis, proteinuria.	Negative	Not completed	Probable (clinical criteria + epidemiologic linkage)	Outpatient oral doxycycline for seven days	Complete recovery
C	20s	F	Icteric. Fever, jaundice, diarrhea.	Not available (patient sought care in Ecuador).	No record	IgM positive, MAT negative	Probable (clinical criteria + presumptive laboratory evidence)	No targeted therapy for leptospirosis	Complete recovery

^#^ Diagnostic classification is documented as per CDC Leptospirosis (*Leptospira interrogans*) 2025 Case Definition [27]. Abbreviations: CXR = chest x-ray (radiograph); AUS = abdominal ultrasound; MAT = microscopic agglutination test; IV = intravenous.

**Table 2:** Differential diagnosis of fever in a returned traveler with freshwater adventure exposure [65,66,67,68,69].

Disease	Organism	Incubation	Geographic Distribution	Vector/Exposure	Clinical Features	Diagnosis	Treatment
Bacterial							
Leptospirosis	*Leptospira* spp.	5–14 days (2–30)	Widespread distribution	Direct or indirect contact with urine from infected animals.	Non-specific febrile illness, myalgia, conjunctival suffusion, jaundice, hemorrhage (pulmonary), acute renal injury, hepatocellular injury, thrombocytopenia, proteinuria. May have biphasic illness.	Serology (IgM and IgG ELISA; Microscopic agglutination test), PCR, culture.	Mild: doxycycline, azithromycin, penicillins Severe: IV third generation cephalosporin, penicillins, doxycycline
Acute bacterial traveler’s diarrhea	*Enterotoxigenic Escherichia coli (ETEC)*, *Shigella* spp., *non-typhoidal Salmonella enterica*, *Campylobacter* spp., rarely reported organisms such as *Aeromonas* and *Pleisiomonas*	6 h–10 days depending on organism	Widespread distribution	Fecal-oral; ingestion of contaminated water.	Watery (ETEC, *Salmonella*) or bloody (*Shigella*, *Campylobacter*) diarrhea, abdominal cramps.	Stool culture with antimicrobial susceptibility testing, where appropriate.	Hydration; antibiotics (fluoroquinolone or azithromycin) depending on organism and severity of illness.
Melioidosis	*Burkholderia pseudomallei*	1–21 days (long, variable incubation period of months-years)	Tropical and subtropical areas, with large foci in SE Asia, Northern Australia, and Indian subcontinent	Inhalation, cutaneous inoculation, or ingestion of infected soil or water.	Most common presentation is acute pneumonia; most severe complication is acute septicemia. Other forms include local cutaneous disease, osteomyelitis, septic arthritis, and abscess in the liver, spleen, kidney, prostate, or parotid gland.	Culture from any suspected site of infection. Serology of limited use.	Intensive phase with carbapenem or ceftazidime, followed by eradication phase with trimethoprim/ Sulfamethoxazole (alternatives are doxycycline or amoxicillin-clavulanic acid).
Tularemia	*Fransicella tularensis*	3–5 days (1–14 days)	Sporadic global foci; most common in North America	Contact with infected animals, ingestion of infected food/water, inhalation, vector-borne (tick, fly, flea bite).	Three major forms: ulceroglandular, pneumonic, typhoidal. Pulse-temperature dissociation.	Culture, PCR, serology.	Doxycycline or tetracycline for mild cases; parenteral streptomycin or gentamicin for severe cases.
*Vibrio vulnificus*	*Vibrio vulnificus*	1–2 days	Warm coastal regions around the globe	Exposure to fresh or brackish waters; raw seafood ingestion.	Gastrointestinal symptoms; pustular and bullous skin lesions, lymphangitis, severe cellulitis, necrotizing fasciitis (frequently bilateral), myositis, gangrene; primary septicemia in 60% of cases.	Culture (blood, wound, stool).	Third generation cephalosporin and doxycycline; surgical debridement for severe soft tissue infections.
Viral							
Hepatitis A	*Hepatitis A virus*	28 days (14–45 days)	Widespread distribution	Fecal-oral; ingestion of contaminated water.	Fever, gastrointestinal symptoms, acute liver injury.	Serology (Hepatitis A IgM).	Supportive management.
Norovirus	*Norovirus*	24–48 h (12–72 h)	Widespread distribution	Fecal-oral; ingestion of contaminated water.	Fever, watery diarrhea, nausea, vomiting, abdominal pain.	RT-qPCR assays, multiplex gastrointestinal viral PCR (MGVP)	Supportive management.
Parasitic							
Cryptosporidiosis	*Cryptosporidium* spp.	7–10 days (3–28 days)	Widespread distribution	Fecal-oral; ingestion of contaminated water.	Diarrhea, often voluminous and watery. Risk factors for prolonged diarrhea include children and immunocompromised.	Stool PCR, O&P microscopy, EIA.	Supportive management, as usually self-limiting. Nitazoxanide for persistent symptoms or immunocomprising conditions.
Giardiasis	*Giardia lamblia*	7–14 days	Widespread distribution	Fecal-oral; ingestion of contaminated water.	Diarrhea, steatorrhea, bloating, abdominal cramps. May have features of malabsorption.	Stool PCR, O&P microscopy, antigen detection.	Metronidazole, tinidazole, nitazoxanide, or albendazole.
Acute schistosomiasis (Katayama Fever)	*Schistosoma* spp. *	2–12 weeks	Africa, South Asia, Southeast Asia, China, Philippines, South America	Freshwater exposure; penetration of skin by infective cercariae.	Fever, urticarial rash, cough, dyspnea, abdominal pain, peripheral eosinophilia, pulmonary infiltrates. Neurological manifestations, including transverse myelitis.	Serology, stool and urine O&P.	Corticosteroids for acute symptom management, followed by Praziquantel once larvae have matured to adult trematodes.
Free-living amoebiasis (Granulomatous amoebic encephalitis (GAE) and Primary amoebic meningo-encephalitis (PAM)).	GAE: *Acanthamoeba* spp., *Balamuthia mandrillaris* PAM: *Naegleria fowleri*	GAE: weeks to months PAM: 1–14 days	Worldwide distribution. *Balamuthia* most commonly reported in southern US and South America.	Contact with freshwater or soil	GAE: low-grade fever, headache, neurological deficits with slow progression to deficits to seizure, coma, and death. PAM: fever, headache, photophobia, alterations of taste and smell, with rapid progression to altered mental status, seizure, focal cranial nerve palsies, coma, and death. Other: keratitis (*Acanthamoeba* spp.), granulomatous skin lesions (*Balamuthia mandrillaris*, less commonly *Acanthamoeba* spp.).	Lumbar puncture with CSF microscopy. Biopsy of brain tissue (for trophozoites and cyst forms). PCR of CSF or brain tissue. Biopsy of skin lesion if present. Biopsy, corneal scrapings, or culture if suspicion of *Acanthamoeba* keratitis.	Combination therapy with regimens including miltefosine, azoles, pentamidine, amphotericin, and other agents. Based on case report data. High-dose steroids used for cases of cerebral edema.
Fungal							
Endemic mycoses (fungal pneumonia)	*Histoplasma capsulatum*, *Blastomyces dermatitidis*	3–17 days (histoplasmosis) 30–45 days (blastomycosis, range 21–106 days)	Endemic in parts of North, Central, and South America	Inhalation	Dry cough, myalgia, headache, respiratory distress.	Respiratory culture and microscopy, PCR, serology. Antigen testing for *Histoplasma*.	Itraconazole for mild-moderate disease; LFAmB for CNS, severe, or disseminated disease.

* *Schistosoma mansoni*, *Schistosoma haematobium*, *Schistosoma japonicum*, *Schistosoma mekongi*, *Schistosoma intercalatum*, *Schistosoma guineensis*.

## Data Availability

All data are presented in the manuscript.
